# WormGUIDES: an interactive single cell developmental atlas and tool for collaborative multidimensional data exploration

**DOI:** 10.1186/s12859-015-0627-8

**Published:** 2015-06-09

**Authors:** Anthony Santella, Raúl Catena, Ismar Kovacevic, Pavak Shah, Zidong Yu, Javier Marquina-Solis, Abhishek Kumar, Yicong Wu, James Schaff, Daniel Colón-Ramos, Hari Shroff, William A. Mohler, Zhirong Bao

**Affiliations:** Developmental Biology Program, Sloan-Kettering Institute, New York, NY USA; Present Address: Institute for Molecular Life Sciences, University of Zürich, Zürich, Switzerland; School of Energy and Power Engineering, Jiangsu University of Science and Technology, Zhenjiang, Jiangsu 212003 China; Program in Cellular Neuroscience, Neurodegeneration and Repair, Department of Cell Biology, Yale University School of Medicine, New Haven, CT USA; Section on High Resolution Optical Imaging, National Institute of Biomedical Imaging and Bioengineering NIH, Bethesda, MD USA; Dept. of Genetics & Dev Biology, University of Connecticut Health Center, Farmington, CT USA; Center for Cell Analysis and Modeling, University of Connecticut Health Center, Farmington, CT USA

**Keywords:** Visualization, *C. elegans*, Morphogenesis, Neurons, Single-cell analysis

## Abstract

**Background:**

Imaging and image analysis advances are yielding increasingly complete and complicated records of cellular events in tissues and whole embryos. The ability to follow hundreds to thousands of cells at the individual level demands a spatio-temporal data infrastructure: tools to assemble and collate knowledge about development spatially in a manner analogous to geographic information systems (GIS). Just as GIS indexes items or events based on their spatio-temporal or 4D location on the Earth these tools would organize knowledge based on location within the tissues or embryos. Developmental processes are highly context-specific, but the complexity of the 4D environment in which they unfold is a barrier to assembling an understanding of any particular process from diverse sources of information. In the same way that GIS aids the understanding and use of geo-located large data sets, software can, with a proper frame of reference, allow large biological data sets to be understood spatially. Intuitive tools are needed to navigate the spatial structure of complex tissue, collate large data sets and existing knowledge with this spatial structure and help users derive hypotheses about developmental mechanisms.

**Results:**

Toward this goal we have developed WormGUIDES, a mobile application that presents a 4D developmental atlas for *Caenorhabditis elegans*. The WormGUIDES mobile app enables users to navigate a 3D model depicting the nuclear positions of all cells in the developing embryo. The identity of each cell can be queried with a tap, and community databases searched for available information about that cell. Information about ancestry, fate and gene expression can be used to label cells and craft customized visualizations that highlight cells as potential players in an event of interest. Scenes are easily saved, shared and published to other WormGUIDES users. The mobile app is available for Android and iOS platforms.

**Conclusion:**

WormGUIDES provides an important tool for examining developmental processes and developing mechanistic hypotheses about their control. Critically, it provides the typical end user with an intuitive interface for developing and sharing custom visualizations of developmental processes. Equally important, because users can select cells based on their position and search for information about them, the app also serves as a spatially organized index into the large body of knowledge available to the *C. elegans* community online. Moreover, the app can be used to create and publish the result of exploration: interactive content that brings other researchers and students directly to the spatio-temporal point of insight. Ultimately the app will incorporate a detailed time lapse record of cell shape, beginning with neurons. This will add the key ability to navigate and understand the developmental events that result in the coordinated and precise emergence of anatomy, particularly the wiring of the nervous system.

## Background

Biologists must increasingly analyze and interpret the behavior of single cells in complex, spatially variant tissue. Microscopy improvements now make it possible to produce highly detailed records of complex structures as they develop. It is possible to perform in toto imaging of metazoan development [1–4] with the resolution necessary to follow individual cells. Increasingly sophisticated stem cell and tissue engineering applications also motivate a need to follow cell movements and tissue morphogenesis at the single cell level. Image analysis algorithm development supports these efforts by allowing increasingly automated distillation of these 4-dimensional datasets into curated records of tracked cells [4–7]. However, manually navigating hundreds of cell tracks over hours is a daunting task. Developmental events often involve large numbers of cells that are inherently embedded in a complex spatio-temporal context. Meaningful information is only implicit in the undifferentiated mass of tracks produced by cell tracking. Software tools have been developed to aid in viewing images along with annotation in order to validate or edit the results of image analysis [8–11]. There remains however, a need for tools focused on navigating this wealth of cell identity and position information in order to see and understand events of interest amidst the clutter of complex unrelated events. In addition, spatio-temporal data about cells does not exist in a void. There is extensive prior knowledge available about developmental events and gene expression that can be better understood when placed in a spatio-temporal context. Systematic analysis of developmental regulation [12] adds to the volume and complexity of this data. Anatomical atlases in adult *C. elegans* [13, 14] and brain atlases in other models e.g., [15, 16] lack temporal information and are usually focused on presenting a single self-contained set of data. Tools are needed to enable the exploration of complex 4D records of multicellular systems alongside contextual data. A spatial data infrastructure for complex tissues would provide a systematic method of exploring and collating developmental information in a unified framework, analogous to the role of GIS and associated data infrastructure in revolutionizing access to and the use of geographical information.

*C. elegans* is an approachable point of entry in attacking these problems of data visualization and interpretation. Popular because of its simplicity, *C. elegans* has an invariant lineage; in each embryo the same number of cells are born and assume the same functions. In every embryo the same cells play out tightly conserved movements and shape changes over the eight hours between fertilization and when the embryo begins to move within the egg shell [17–19]. At around 7 h, the comma stage, elongation begins and a tail becomes visible. Uniquely, the adult neural structure has been mapped out at the synaptic level [20], meaning the end-state of neural developmental processes is known. The 670 embryonic cell divisions, 113 programmed cell deaths, and 302 neurons that go into an adult worm provide a fixed landscape on which developmental data can be superimposed. In addition, since an embryonic cell exists over a relatively short period of time, embryonic cell names provide an approximate 4D location, making it possible to connect existing and new knowledge to the spatio-temporal context of that individual cell.

The goal of WormGUIDES is to enable the visual exploration of this information by providing an interface for examining single-cell level records of development. The invariant lineage of C. *elegans* allows these records to become a spatial index into community knowledge bases. Detailed positional information is thus linked to community knowledge: gene expression, web pages and databases. This portal creates an easy-to-navigate and more unified view of development. Although the adult neural structure of *C. elegans* is known, and full time-lapse records of cell positions are available, much less is understood about how developing neurons coordinate position and growth to generate adult neural structures. The expectation is that easy visualization, customization, and spatial search will enable a deeper understanding of how complex multicellular processes, especially neural development, unfold in a spatial context. These general tools will be critical not only in *C. elegans* but across all organisms and experimental systems where complex developmental processes unfold.

## Implementation

The WormGUIDES mobile app was developed to address the need for data exploration in 4D datasets. Developed in Java for Android and Objective-C for iOS, WormGUIDES is open source, with the source code available at http://www.wormguides.org/open-source-software (Android) and https://bitbucket.org/raulcatena/iworm/ (iOS). For the end user, the app and underlying data can be downloaded and installed for free through the Google Play Store and Apple App Store.

**Fig. 1:**
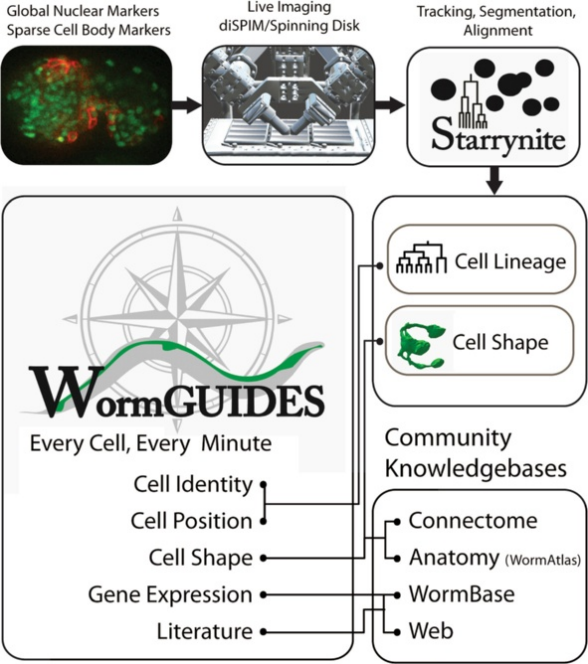
WormGUIDES vision and input data. The goal of WormGUIDES is to enable novel visual exploration of development by providing an intuitive interface for exploring single cell level records of embryonic development in 4D. WormGUIDES also provides a spatial index into community knowledge bases by enabling users to quickly search for information on cells they have identified via 4D exploration. Detailed cell positions and (in future releases) cell morphology are combined with available knowledge to create a convenient way to navigate views of development. The expectation is that this ability to understand cells in the context of the embryo will enable a deeper understanding of how complex processes, particularly neural development, unfold

Figure 1 outlines the information collected into WormGUIDES. The core of WormGUIDES is a record of the location of every cell at every minute during embryonic development. This reference model has been built from the measured nuclear positions of every cell in an individual hemaphrodite embryo between the four cell and comma stage with 599 tracked cells (including dead cells that remain visible) in the last frame. Cell tracking was performed with Starrynite [5, 21]. Cell tracking results were edited and validated via systematic manual comparison with images, and automated comparison of cell positions measured in 2 additional embryos to flag positional outliers as potential errors for manual inspection.

This detailed record of development is complemented by extensive pre-existing knowledge regarding fate and gene expression. A wide range of information is available online and through *C. elegans* specific websites. Embryonic cell names provide a way to spatially index this information and link to it from the WormGUIDES app. Key information about cell fate and gene expression is integrated into the app directly. The fate of each cell in the C. *elegans* embryo is known, and this knowledge is summarized in the standard parts list [22]. Partial single cell level expression information is available for many genes through the WormBase [23] website. Gene expression information is an ideal example of information that, although inherently spatio-temporal, is typically not presented that way. Rather, it has been available as curated lists of cell names with known expression. WormGUIDES contains the detailed positions of these cells over their lifetimes and this information has been integrated to enable the exploration of the spatio-temporal distribution of expression. Valuable insights into the control of a process can be gained by cross referencing expression with the stages of the process, allowing users to easily screen known molecular actors that might contribute to the event of interest.

**Fig. 2:**
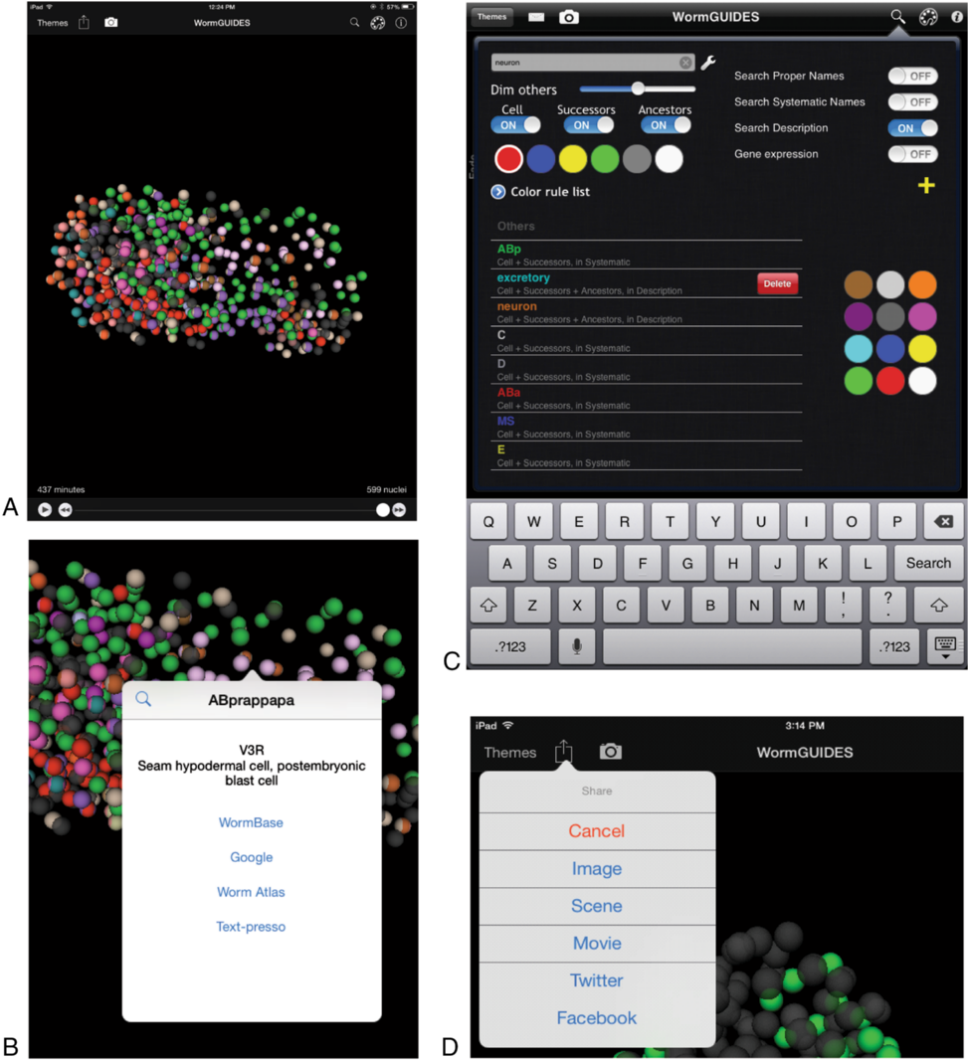
WormGUIDES app user interface. **a**. Interface overview showing the interactive nuclear position model with cells colored by tissue fate. **b**. Information pop up that appears upon tapping on a cell. This pop up shows the cell name. Upon expanding it with a tap the fate description of terminal cells is displayed. The cell can be recolored by tapping the magnifying glass,or a search for that cell can be executed against online knowledgebases. **c**. The search interface allows users to intuitively control the 3D model’s colors. Text searches can be executed against systematic names, terminal cell names and fate descriptions as well as online searches against WormBase gene expression information. **d**. The sharing panel which allows the user to share a screen shot or a scene definition that can be loaded by other app users

The app presents nucleus position and identity over time in a multi-touch interface (Fig. 2a). Developmental time is controlled with a scroll bar, while spatial navigation is controlled by standard dragging and pinching gestures. When a feature of interest is identified, surrounding cells can be queried for their identity. Tapping on any cell displays its systematic name in a pop up dialog. For terminal cells, a functional name and parts list description are also displayed (Fig. 2b). Options in the pop up allow several community specific websites to be queried as well as general web searches to be performed for the selected cell’s name. This allows the app to serve as an aggregate spatial index of existing knowledge about single-cells during *C. elegans* development.

A key part of the app interface is the search panel (Fig. 2c). The goal of this panel is to allow easy composition of complex visualizations that highlight specific subsets of cells. As an example, the color scheme in Fig. 2a colors terminal cells based on their fate. Colors follow the tissue color conventions of WormAtlas [24], a community anatomy knowledge base. Sliders explicitly control the type of search executed: systematic names, functional cell names, parts list descriptions or gene expression search. Labels can be propagated to the ancestors and descendants of these cells as well. The resulting search applies a new color to the cells that match the search terms. Multiple searches can be layered to highlight these cellular features within the global context of the entire embryo.

The scene sharing menu is designed to facilitate communication between researchers (Fig. 2d). Once a custom visualization has been created a user can share it with others. This can be done by sending a screen shot by email, or saving the screen shot as a figure for reference. Alternatively, and most powerfully, the WormGUIDES scene configuration can be shared as a URL that encodes the coloring and camera position in the scene. This allows other app users to generate their own fully configurable instance of the original visualization within the WormGUIDES app.

## Results and Discussion

**Fig. 3:**
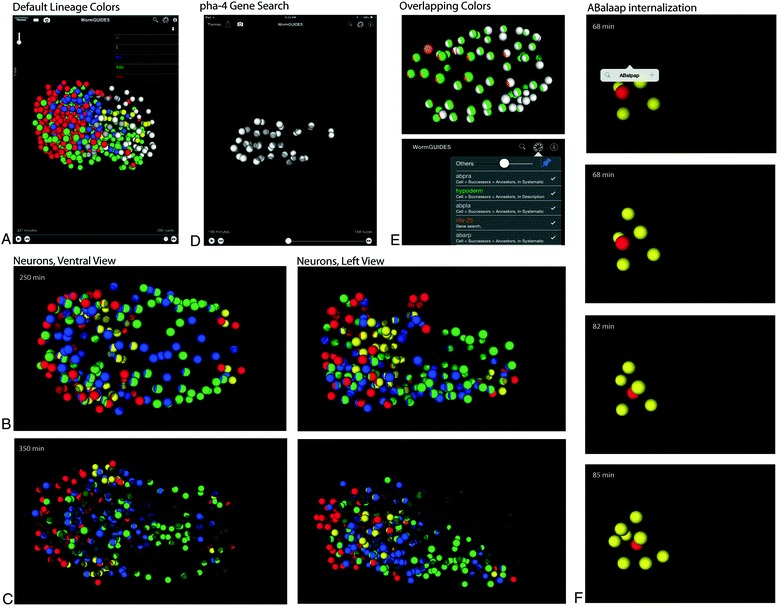
Customizing visualization with the search inteface. **a**. A lineage color scheme with each founder cell lineage in a different color. **b**. Early ~500 cell view of embryo highlighting neuronal subtypes from left and ventral views. **c**. A view of the same color scheme, approximately two hours later showing rearrangement of cells into more tightly organized neural tissue. **d**. Results of a gene search for pha-4 showing the pharynx primordium and gut cells. **e**. Overlapping colored sublineages, each highlighting hypodermal fate using a different method, and a close up of the color key corresponding to the embyro illustrate how overlapping color schemes are rendered by striping the colors that apply to a cell

We demonstrate the use of the WormGUIDES app by creating visualizations highlighting the key features of the app and their ability to highlight important biological structures. The default color scheme in the app is lineage based (Fig. 3a). Each of six founder cells is assigned a different color which is applied to all its descendants, thus coloring the embryo based on clonal descent. Searching based on cell fate simplifies highlighting key structures and their arrangement at particular developmental stages. Coloring a set of cells with a given fate allows the arrangement and relative positions of these cells to be followed over time. Neuronal subtypes and ancestors are highlighted in Fig. 3b, motor neurons in green, sheath and socket cells in red, interneurons in blue and sensory neurons in yellow. Each category was highlighted by performing a keyword search against part list descriptions. The cells at comma stage have a distinct spatial arrangement with a clear correspondence to the final structures, such as the ventral cord (central posterior cluster in green); about two hours earlier the structures are significantly less distinct (Fig. 3c). Neighboring cells, potential players in events, can be queried for their names with a tap.

Gene expression (Fig. 3d) based coloring is a key instance of integrating external information into a unified spatial framework. When a gene search is executed, a query is made for the WormBase page for that gene and the expression field on the page is parsed for a list of embryonic cell names. These cells are used as the search result. Results often give a useful sense of early embryonic expression. The results of a pha-4 search, for example, appropriately color the pharynx primordium and the gut cells. Drawing on live data means results will inherently reflect updates as more systematic gene expression information [25] is integrated into WormBase. This transplantation of expression information back into a spatial context is possible because of the fixed spatial relationships between cells in *C. elegans*, and provides a detailed, interactive visualization that would be all but impossible without the benefit of WormGUIDES.

Each search result can be seen as a layer of information visualized as colors. In general layers from different results may overlap. This is indicated in the app by striping nuclei with all colors that apply to that cell. Overlapping search results in Fig. 3e illustrate striping. Hypodermal cells are colored with three distinct search methods: part list description search for the term ‘hypodermal’, systematic name search for 3 sub-lineages that are heavily hypodermal and gene search for nhr-25 associated with hypodermal fate. Overlap, or its lack, between these searches can be easily observed and used to guide investigation of individual cell identities.

### Future work

Significant additions to WormGUIDES functionality are planned. A key priority is a desktop version of WormGUIDES. This will allow browsing of raw image data as well as cellular morphology and take advantage of additional screen space to provide more sophisticated tools for data exploration. Additional windows and data management tools will make it possible to view and manipulate online search results and use them to control visualization in more flexible ways. Another key feature of the desktop version will be the ability to add and navigate arbitrary user generated annotations of events or structures either manually or by importing data tagged with cell names or spatio-temporal positions.

**Fig. 4:**
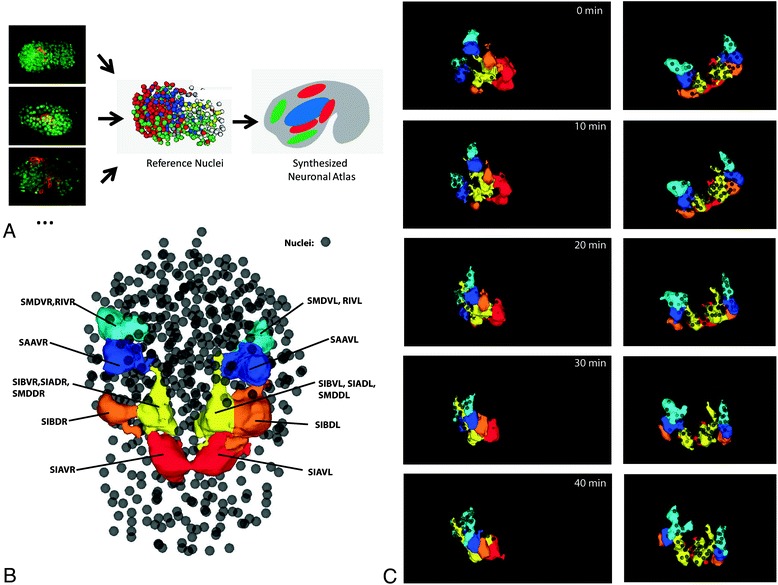
Toward integration of neural morphology into WormGUIDES. **a**. Schematic of the neuron shape characterization strategy. Multiple strains with different subsets of labeled neurons are lineaged and segmented. Nuclear positions are used to align data to a single reference coordinate system. **b**. Key to *lim-4* expressing cells identities. **c**. A time lapse 3D reconstruction of pairs of left right symmetric neurons expressing *lim-4* imaged using a diSPIM system. Close, difficult to resolve clusters of cells are segmented as a single object. Nuclei are rendered as small gray spheres. Cell bodies are colored to show left right symmetry. A series of images of the reconstruction spanning 40 min is shown. Time 0 is an arbitrary point where *lim-4* expression is clearly visible; Time 40 is approximately 10 min before twitching begins. Cell lineage and cell shape are semi-automatically segmented and tracked

The gradual addition of cell morphology data to WormGUIDES a major focus of ongoing efforts. Fig. 4 briefly presents the strategy for characterizing cell morphology and some preliminary results toward this goal. In the short term, the morphology of neuronal cells is our focus, though our approach, and ultimate ambition, is general. Our strategy (Fig. 4a) is to analyze embryos from a number of strains, each of which uses promoters to label different subsets of neurons. This sparse labeling aids clear time-lapse imaging, and greatly simplifies the segmentation of cell shape. Nuclear positions are tracked in each embryo and these are used to align results to a unified coordinate system allowing a synthesized atlas of neuronal shape to be created.

Figure 4b,c demonstrates this process for one strain labeled with *lim-4*::GFP (mgIs19[lim-4::gfp, pRF4]; ujIs113 [Ppie-1::H2B::mCherry, Pnhr-2::HIS-24::mCherry, unc-119(+)]). At the comma stage *lim-4*::GFP labels at least 8 pairs of left-right symmetric cells that are part of the nerve ring. Cell identities (Fig. 4b) were established by lineaging using Starrynite. Time lapse data of cell bodies were acquired with a Dual-view Inverted Selective Plane Illumination Microscopy (diSPIM) system [26, 27] and segmented semi-automatically in Imaris based on image intensity. *lim-4*::GFP expressing cells were tracked, temporal alignment was manually assigned and cell positions in each frame were used to compute a best fit (rigid plus scaling) transformation between the *lim-4*::GFP embryo and WormGUIDES nuclear positions. Fig. 4c shows the *lim-4*::GFP cells superimposed on the WormGUIDES embryo in a series of frames covering 40 min of development before twitching commences. Nuclei are rendered as small semi-transparent spheres to avoid obscuring the cell bodies. Over the sequence of frames it is possible to see the bundle of processes that make up the nerve ring extending dorsally.

As the *lim-4*::GFP proof of concept data suggests, neural cell morphology integrated into the app will add an extra dimension of information, highlighting when changes in morphology occur, and the sequence of interactions that assemble cells into the structures revealed by the adult wiring diagram. Ongoing work will scale up the processing of neuronal markers, including automating membrane marker segmentation [28], and finalize algorithmic details of assembling these data into a unified atlas.

Alignment of data from many embryos will require a more systematic understanding of variability in cell position over time. Previous systematic study of the embryo up to the 350 cell stage suggests it is reasonable to represent nuclear position as unimodal, with limited variability [29]. Collection of additional late stage data to assess variability is in progress. Ultimately, cell positions within the app that are based on a single embryo may be replaced by an aggregate consensus model. An explicit representation of observed variability is a likely feature of the desktop atlas. Eventual analysis and integration of a male worm is also possible.

Additional alignment issues will need to be addressed to characterize post-twitching development, which can be imaged using diSPIM but will require the development of methods for straightening and aligning twisted embryos. The tightly packed elongated embryo presents a more difficult problem than the normal curved pose of the adult worm [30]. Methods are under development to straighten the embryo using a combination of junctional and nuclear markers to semi-automatically extract a 3D model of the worm’s body in each frame and unwrap it into a consistent straightened coordinate system [31].

## Conclusions

The WormGUIDES app provides users with the ability to explore and navigate developmental events in their spatio-temporal context and cross reference these events against the accumulated knowledge amassed by the worm community. By simplifying exploration of the developing embryo, WormGUIDES should make it easier to understand the coordinated motions of cells as they unfold in a spatio-temporal context. This in turn should make it easier to correlate these events with gene expression and other information in order to develop testable hypotheses about mechanistic control of development. The visualization customization interface within the app allows any user to investigate embryonic events they care about, regardless of what those events are. As it stands, the WormGUIDES app provides a useful tool for navigating development in complex tissue and examining the coordinated behavior of cells over time. We hope it will find use as both a reference for the study of *C. elegans* development and as an educational tool. These applications are not unique to the worm community. The kinds of knowledge available to the worm community are becoming increasingly standard as the availability of genomic and other big data increases for other systems. Spatially localizing this information will become possible in other models as paradigms develop for handling cell-level correspondence between individuals in organisms with variable cell lineages [32]. The fundamental types of data, interactive tasks and challenges of alignment to reference data addressed by WormGUIDES are universal and our solutions should be applicable to other organisms.

## Availability and requirements

Project name: WormGUIDESProject home page: http://www.wormguides.org/ Operating systems: Android, iOSProgramming languages: Java, Objective-CLicense: GNU GPL
